# Stimulus Intensity Affects Variability of Motor Evoked Responses of the Non-Paretic, but Not Paretic Tibialis Anterior Muscle in Stroke

**DOI:** 10.3390/brainsci10050297

**Published:** 2020-05-15

**Authors:** Anjali Sivaramakrishnan, Sangeetha Madhavan

**Affiliations:** 1 Brain Plasticity Lab, Department of Physical Therapy, College of Applied Health Sciences, University of Illinois at Chicago (UIC), Chicago, IL 60612, USA; asivar5@uic.edu; 2 Graduate Program in Rehabilitation Sciences, College of Applied Health Sciences, University of Illinois at Chicago, Chicago, IL 60612, USA

**Keywords:** motor evoked potential, stimulus response curve, lower limb, variability, stroke

## Abstract

Background: Transcranial magnetic stimulus induced motor evoked potentials (MEPs) are quantified either with a single suprathreshold stimulus or using a stimulus response curve. Here, we explored variability in MEPs influenced by different stimulus intensities for the tibialis anterior muscle in stroke. Methods: MEPs for the paretic and non-paretic tibialis anterior (TA) muscle representations were collected from 26 participants with stroke at seven intensities. Variability of MEP parameters was examined with coefficients of variation (CV). Results: CV for the non-paretic TA MEP amplitude and area was significantly lower at 130% and 140% active motor threshold (AMT). CV for the paretic TA MEP amplitude and area did not vary with intensity. CV of MEP latency decreased with higher intensities for both muscles. CV of the silent period decreased with higher intensity for the non-paretic TA, but was in reverse for the paretic TA. Conclusion: We recommend a stimulus intensity of greater than 130% AMT to reduce variability for the non-paretic TA. The stimulus intensity did not affect the MEP variability of the paretic TA. Variability of MEPs is affected by intensity and side tested (paretic and non-paretic), suggesting careful selection of experimental parameters for testing.

## 1. Introduction

Transcranial magnetic stimulation (TMS) induced motor evoked potentials (MEP) are widely used as a neurophysiological biomarker to assess corticomotor excitability of the central nervous system and determine the magnitude of neurophysiological change induced with motor practice or training. Common approaches for quantifying contralateral descending corticomotor drive include either plotting a stimulus-response (SR) curve over a range of stimulation outputs or choosing a single TMS stimulator intensity.

Although the MEP is recognized as a sensitive neurophysiological measure, there is considerable inter- and intra-stimulus variability, an issue that affects data interpretation [1]. Variability of MEPs limits the ability to fully attribute changes in corticomotor drive to the effects of an intervention or to a dysfunctional state of the motor system. MEP variability can be influenced by several intrinsic and extrinsic factors. Some intrinsic factors include fluctuations in neuronal excitability [2], physiological noise [3], muscle activation level [4], attention [5], time of day [6], age, and neuronal motor threshold [7]. Extrinsic factors affecting variability can include technical factors such as TMS coil location and placement [1], stochastic noise, environmental conditions, experimenter skill levels, and measurement bias [8]. Obtaining a complete recruitment curve may provide a better estimate about the gain of the corticomotor system and reduce variability associated with measurement at individual stimuli [9]. However, experimental time constraints can preclude recording of SR curves, which are labor- and time-intensive and create a larger participant burden than measurements at single intensities. Reducing technical constraints for TMS is necessary for it to be potentially integrated into clinical practice [10].

A minimum of five MEPs to an ideal number of 30 has been suggested to obtain a reliable estimate of MEP amplitude [11,12,13,14,15]. However, there is still no consensus regarding the ideal stimulus intensity when choosing to test only at a point estimate. Previously, lower limb reliability studies in healthy individuals and stroke have used a threshold criterion, where the intensity is adjusted to the individual’s resting motor threshold (RMT) or active motor threshold (AMT), usually set around 120% RMT or AMT [11,16,17]. The 120% intensity criterion appears to be arbitrary and, to our knowledge, we have not come across any study that establishes precedence for choosing 120% over other intensities. Recent guidelines for recording MEPs recommend that an optimal intensity should correspond to the transition zone from the rising slope to the plateau portion of the SR curve, approximately 140% of RMT or 170% of AMT [18]. These guidelines are mostly based on TMS measurements in the upper-limb muscles. Despite the growing interest in examining lower limb corticomotor connectivity, data from lower limb studies are limited. Typically, lower limb muscles are tested using the double cone coil, which provides diffuse, but deeper penetrating currents, and intensities above 80% maximum stimulator output (MSO) are not well tolerated. While point sampling at an intensity of 170% AMT is feasible for upper limb muscles (where studies use a figure of eight or flat coil) and healthy brains (where thresholds are lower), individuals with stroke have elevated thresholds for the leg muscles and, in our experience, participants find higher intensities (above 80% MSO with the double cone coil) uncomfortable. It is also important that the chosen test intensity demonstrates reduced variability. Few studies have elucidated the relationship between MEP variability and stimulus intensity for leg muscles in stroke. Beaulieu et al. (2017) reported that motor threshold and MEP latency (at 120% AMT) had lower measurement error than other MEP parameters for the tibialis anterior (TA) muscle in stroke [17]. Cacchio et al. (2011) reported lower measurement error for the non-paretic TA compared with the paretic TA for motor threshold, MEP latency, and amplitude (at 100% AMT) [19]. However, it is still unclear how test intensity influences MEP variability for the lower limb in individuals with stroke. This is important to understand as several stroke related studies prefer to report MEPs recorded at single test intensities as indices for measuring recovery or use it as diagnostic/prognostic tool. Moreover, there is no consensus for selecting the test intensity, which is important to consider when comparing results across several studies. Therefore, in this exploratory study, we chose to examine variability of MEP parameters for the paretic and non-paretic TA muscle for determining the optimal stimulus intensity with the least variability in individuals with chronic stroke.

## 2. Materials and Methods

We retrospectively analyzed baseline TMS data obtained during a larger randomized controlled trial (RCT) (clinical trial registration: NCT03492229) conducted in the Brain Plasticity Laboratory at the University of Illinois at Chicago. Individuals with a single, unilateral stroke (>6 months from onset), age 50–80 years, with residual gait deficits and ability to walk without an ankle orthosis for 5 min at comfortable pace were included in the clinical trial. Our exclusion criteria included presence of severe osteoporosis, contractures in the lower limb, consumption of anti-spasticity medications, presence of cardiorespiratory or metabolic disorders or any infectious disease and contraindications to TMS such as presence of metal implants in the brain, a history of seizures and medications that alter central nervous system excitability, a history of skull fracture and/or concussion, and brainstem/cerebellar lesions or the presence of cognitive impairments. In addition, data from individuals with no detectable MEPs in the paretic TA muscle were excluded. All participants in the RCT provided written, informed consent and the experimental protocol was approved by the Institutional Review Board at University of Illinois at Chicago (#2011-0676, date of approval-11/3/2011) and adhered to the Declaration of Helsinki.

### 2.1. Electromyography (EMG)

EMG data were collected from the paretic TA (PTA) and non-paretic TA (NPTA) muscles using surface Ag/AgCl electrodes that were placed over the muscle belly of the respective muscles [20]. The ground electrode was placed over the spinous process of the seventh cervical vertebra. EMG data were sampled at 2000 Hz, amplified (1000×) and band pass filtered (10–500 Hz) with a EMG system (Bagnoli 8, Delsys, Natick, MA, USA). Spike2 software (version 7.2, Cambridge Electronic design, Cambridge, UK) was used to collect and analyze EMG data. Maximum voluntary contraction (MVC) data were obtained using the maximal value of three trials for both muscles.

### 2.2. TMS

Single pulse TMS was administered with a 110 mm double cone coil (posterior–anterior orientation) connected to a Magstim 200 stimulator (Magstim, Dyfed, Wales, UK). Participants were provided visual feedback of muscle activity and required to maintain a tonic contraction corresponding to approximately 10% MVC during MEP recordings. The hotspot for the TA muscle was determined according to techniques that we have reported previously [21]. We defined the active motor threshold (AMT) as the stimulus intensity resulting in identifiable MEPs (≥0.1 mV) in 50% of eight trials each from the target TA muscle. We recorded MEPs for intensities ranging from 80% to 140% AMT (seven intensities, six stimuli at each intensity, random order) for the PTA and NPTA muscles. We chose to record six stimuli at each intensity, as a recent systematic review and meta-analysis by Cavaleri et al. (2017) concluded that a minimum of five stimuli can produce reliable MEP recordings for a single session study [14]. This is also supported by data from Lewis et al. (2014), who reported that the reliability of soleus MEPs for a single session was excellent with six stimuli [11]. As we were collecting MEPs at seven different intensities from two muscles (total of 84 stimuli, not counting stimuli used for hotspot and threshold measurements), increasing the number of stimuli per intensity any further will produce a great burden on participants in addition to mental/physical fatigue, which may also influence results. The inter-stimulus interval in our study was approximately 4 s to minimize carry over effects of the previous stimuli [22]. Stimulus intensities were delivered in a random order and step increments occurred from 3% to 6% MSO, corresponding to 10% AMT.

### 2.3. Data and Statistical Analyses

#### 2.3.1. Data Analyses

TMS induced MEPs were quantified using four calculated measures- peak – to - peak amplitude, rectified area, onset latency, and cortical silent period (CSP). A MEP window was established for each muscle by finding the onset and offset latencies [23]. Onset latency (ms) was calculated as the time from the trigger/stimulus artifact to onset of the MEP, identified as the first rise in amplitude above 25% background EMG lasting for at least 5 ms. Amplitude (mV) was calculated as the voltage difference between the maximum positive and maximum negative peaks. Area (mVs) was calculated as the area under the curve of the rectified MEP. CSP (ms) was calculated as the duration from MEP offset to recurrence of background EMG activity above 25% of background EMG. MEP offset was defined as the drop in MEP amplitude below 25% background EMG lasting for at least 5 ms. MEP amplitude, area, latency, and silent period parameters were calculated from 100% to 140% AMT, as it is difficult to identify accurate onset and offset windows at subthreshold intensities.

#### 2.3.2. Statistical Analyses

We computed the coefficient of variation (CV = standard deviation (SD)/mean) for suprathreshold stimulus intensities using the six trials collected at each intensity. CVs were calculated for MEP_100_, MEP_110_, MEP_120_, MEP_130_, and MEP_140_ for the respective intensities corresponding to 100% AMT to 140% AMT for amplitude, area, latency, and silent period. Lower CVs suggest greater precision of the measure and higher CVs suggest greater dispersion of data around the mean [24]. CV data were analyzed with a two-factor repeated measures analysis of variance (rANOVA) with stimulation intensities (five levels: MEP_100_–MEP_140_) and side (two levels: paretic, non-paretic) as within-subject factors. Separate rANOVA tests were performed for amplitude, area, latency, and silent period. When the main effects were significant, multiple comparisons were performed with paired t-tests adjusted with Bonferroni corrections. Effect sizes for each parameter were calculated as partial eta squared statistic (η*_p_*^2^). Effect sizes were classified as small (0.01), medium (0.06), and large (0.14) [25,26]. All tests were two-tailed, and the level of significance was set at 0.05.

## 3. Results

**Figure 1:**
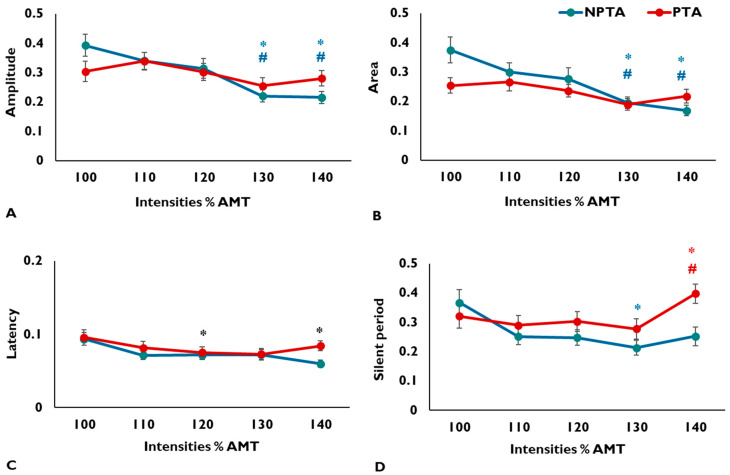
Variability in motor evoked potential amplitude (**A**), area (**B**), latency (**C**), and silent period duration (**D**) parameters as a function of stimulus intensity. The x-axes depict stimulus intensity expressed as % active motor threshold (AMT). The y-axes depict coefficients of variation (CV) for the non-paretic tibialis anterior (NPTA; teal) and paretic tibialis anterior (PTA; red) muscles. Significant interactions were noted for motor evoked potential (MEP) amplitude, area, and silent period, while MEP latency showed a significant main effect of intensity. Teal asterisk denotes CV significantly different from 100% AMT and teal hash symbol denotes CV significantly different from 110 for the NPTA. Red asterisk denotes CV significantly different from 120% AMT and red hash symbol denotes significantly different from 130% AMT for the PTA. Black asterisk denotes significantly different from 100% AMT (main effect of intensity). Data represent averages and error bars represent standard error.

Recruitment curves and stimulation parameters were calculated for 26 participants with stroke (demographics in Table 1). Baseline neurophysiological data for all intensities are shown in Table 2. The CVs for all MEP parameters for the PTA and NPTA are visually demonstrated in Figure 1.

### 3.1. MEP Amplitude

The rANOVA for amplitude revealed a significant intensity by side interaction (F (2.69, 67.32) = 2.82, *p* = 0.050, η*_p_*^2^ = 0.102) (Figure 1A). Post hoc analyses for simple main effects showed that NPTA amplitude CV was significantly higher at MEP_100_ compared with MEP_130_ (*p* = 0.002) and MEP_140_ (*p* = 0.007). Amplitude CV was also significantly higher at MEP_110_ compared with MEP_130_ (*p* = 0.010) and MEP_140_ (*p* = 0.006). Post hoc tests for the PTA were not statistically significant.

### 3.2. MEP Area

The rANOVA for area also showed a significant intensity by side interaction (F (2.6, 66.5) = 2.93, *p* = 0.046, η*_p_*^2^ = 0.105) (Figure 1B). Post hoc tests for simple main effects showed that area CV was significantly higher for the NPTA at MEP_100_ compared with MEP_130_ (*p* = 0.006) and MEP_140_ (*p* = 0.003). Area CV was also significantly higher at MEP_110_ compared with MEP_130_ (*p* = 0.049) and MEP_140_ (*p* = 0.012). Post hoc tests for the PTA were not statistically significant.

### 3.3. MEP Latency

CV for latency showed a significant main effect of intensity (F (4, 100) = 4.70, *p* = 0.002, η*_p_*^2^ = 0.158), but no effect of side or intensity by side interaction (*p* > 0.05) (Figure 1C). Post hoc tests showed that latency CV was significantly higher at MEP_100_ compared with MEP_120_ (*p* = 0.039) and MEP_140_ (*p* = 0.006).

### 3.4. CSP Duration

CV for CSP duration showed a significant intensity by side interaction (F (4, 100) = 2.85, *p* = 0.027, η*_p_*^2^ = 0.103) (Figure 1D). Post hoc tests for simple main effects showed that NPTA silent period CV was significantly higher at MEP_100_ compared with MEP_130_ (*p* = 0.015). For PTA, silent period CV was significantly higher at MEP_140_ compared with MEP_120_ (*p* = 0.034) and MEP_130_ (*p* = 0.011).

## 4. Discussion

The main findings of this study are that variability for MEP amplitude and area for the NPTA was lower at higher stimulus intensities (130 and 140% AMT), but variability for PTA MEP amplitude and area was not affected by intensity. We also noted that MEP latency for both muscles showed the lowest variability at higher intensities. Interestingly, variability in CSP duration was lower at a higher intensity for the NPTA, but increased at higher intensities for the PTA.

### 4.1. Variability in MEP Amplitude and Area

Our findings are congruent with previous studies that show that MEP amplitude and area at higher TMS intensities are less variable [1,7,27,28]. These studies have reported MEP variability for the first dorsal interosseous (FDI) muscle [1,7] and TA muscle [28] in healthy individuals. Only one study in stroke reported a similar finding for the FDI muscle [27]. Although the result that variability of MEP decreases with higher intensity is not novel, this is the first study to confirm these results for the TA muscle over a range of intensities in stroke. It is also likely that the CV would decrease with a further increase in intensity. In our experience, we have found that intensities greater than 140% AMT with the double cone coil (which correspond to approximately 70% MSO and higher for the PTA) is not well tolerated by participants with stroke. Lower variability at higher intensities may be attributed to several factors. At the higher intensities, muscle fibers are maximally activated, which may explain the lower variability. Responses obtained at the rising stage of the recruitment curve are highly dependent on the stimulus intensity, which is influenced by factors such as consistency in coil positioning, location, and tilt of the coil [29]. It is also possible that the nature of the descending volleys elicited by TMS affects variability at higher intensities. Epidural recordings of MEPs have shown that low TMS intensities typically produce an indirect trans-synaptic activation of the corticospinal pathways (I-waves) and higher TMS intensities directly activate the corticospinal tract (CST) neurons (D-waves) [30,31]. The descending I-wave volley is reported to be less synchronous at lower intensities (explaining greater variability) and more synchronous at higher intensities, with a strong D-wave volley [7] and selective firing of the fast conducting CST fibers. We were surprised to observe that MEP variability did not decrease with the increase in stimulus intensity for the paretic muscle. A recent study reported that individuals with higher CST damage showed greater ipsilesional MEP variability at 150% RMT for the FDI muscle [27]. In the present study, we speculate that a decrease in variability with increasing stimulus intensity was not observed for the PTA, as the fast conducting fibers were less likely to be stimulated at higher intensities, because of possible damage by the stroke [27]. The recruitment pattern for the PTA could have also changed at MEP_140_, possibly involving less synchronous I-waves along with a relatively weaker D-wave volley. It is also possible that, at higher stimulus intensities, other functionally redundant motor pathways such as the corticoreticulospinal tracts [32] may be activated [33] and/or ipsilateral descending pathways via the opposite hemisphere may be stimulated owing to current spreading from the coil, affecting variability in muscle responses. Therefore, our findings suggest that using intensities greater than 130% AMT for the NPTA would result in lower variability. Future studies are needed to identify factors such as muscle activation level, age, gender, spasticity, and CST integrity that may affect variability of PTA responses.

### 4.2. Variability in MEP Latency and Silent Period

MEP latency appears to show a trend for lower variability compared with all other parameters in our study, a finding also confirmed by previous studies in healthy individuals and those with stroke [1,17,34,35]. While Beaulieu et al. (2017) report low measurement error at 120% AMT in stroke, here, we show that MEP latency had the lowest CV for all intensities from 100% to 140% AMT [17]. Variability in MEP latency has been explained by the arrival timings of the afferent corticospinal volleys (D- and I-waves) to the spinal motoneurons [36]. Previous reports suggest that MEPs with the shortest latency (higher intensity) are likely induced with D-waves or the direct, monosynaptic motor pathways [37]. This may explain why the overall variability reduced from MEP_100_ to MEP_140_. However, it is unclear why MEP variability reduced from MEP_100_ to MEP_120_, but not from MEP_100_ to MEP_130_.

Interestingly, for CSP duration, we found opposite trends for the NPTA and PTA with respect to stimulus intensity. For the NPTA, variability in CSP duration decreased with increasing intensities, while for the PTA, it increased with intensity. Few studies have reported linear associations between CSP duration and MEP amplitude [38], and CSP duration and stimulus intensity [39], suggesting that CSP duration may be related to the corticospinal outflow that determines the MEP. This may partly explain the contrasting trend in CSP duration variability with increasing intensity for the NPTA and PTA in our study.

### 4.3. Study Limitations

We would like to acknowledge several limitations that may limit the interpretation of our results. Our study is limited by the lack of a repeated measures design, which would allow for further characterization of intra-individual and inter-session variability. It is also important to examine variability with a larger number of stimuli per intensity than currently used in our study. Findings from this study cannot be generalized to a broader cohort of stroke survivors, as we included individuals with elicitable MEPs only. We did not include other imaging measures to characterize CST integrity, which may enable a greater understanding of MEP variability. Finally, our low sample and tested muscle may also affect our generalizability.

## 5. Conclusions

This study provides new information for variability in MEP parameters over different stimulus intensities in participants with stroke. We report that variability of MEP amplitude and area significantly decreases with higher stimulus intensity for the non-paretic muscle. Variability for MEP amplitude and area for the paretic muscle was not affected with stimulus intensity. Moreover, we report, for the first time, variability for MEP latency and silent period as a function of stimulus intensity in stroke. In situations where constructing a SR curve is not feasible, we suggest using test stimulus intensities above 130% AMT to reduce variability and enabling a quick TMS setup to evaluate neurophysiological integrity in the clinic as a possible diagnostic tool. In addition to variability data, we also provide average measurements for several MEP parameters corresponding to stimulus intensities for the NPTA and PTA, which can provide a frame of reference for TMS users performing laboratory or clinical studies for the TA muscle in stroke.

## Figures and Tables

**Table 1:** Demographics.

Age (years)	60.76 (10.28)
Sex (female, *n*, %)	8, 30.76%
Affected side (right, *n*, %)	11, 42.3%
Type of stroke (I/H) *	14/9
Time since stroke (years)	5.05 (4.28)
FMLE (paretic)	24.26 (3.07)
Gait speed (self-selected) (m/s)	0.78 (0.21)

Abbreviations: I; ischemic, H; hemorrhagic, FMLE; Fugl–Meyer lower extremity. Values are expressed as mean (SD) or count (%). * Data for type of stroke were not available for three participants.

**Table 2:** Neurophysiological parameters for non-paretic tibialis anterior (NPTA) and paretic tibialis anterior (PTA).

Intensities (%AMT)	Amplitude (mV)	Area (mVs)	Latency (ms)	CSP (ms)	Intensities (% MSO)
**Non-paretic tibialis anterior**					
100	0.31 ± 0.1	1.77 ± 0.8	29.02 ± 2.4	69.66 ± 45.8	39.54 ± 8.1
110	0.45 ± 0.2	2.72 ± 1.5	28.03 ± 3.1	88.88 ± 51.2	43.50 ± 8.8
120	0.62 ± 0.2	3.93 ± 1.9	28.02 ± 2.3	98.86 ± 48.1	47.46 ± 9.5
130	0.75 ± 0.3	5.07 ± 2.6	27.19 ± 3.0	111.25 ± 55.2	51.42 ± 10.3
140	0.81 ± 0.3	5.41 ± 2.7	27.41 ± 3.3	109.46 ± 54.2	55.38 ± 11.0
**Paretic tibialis anterior**					
100	0.19 ± 0.1	1.81 ± 0.8	32.01 ± 4.8	107.77 ± 74.5	48.88 ± 10.7
110	0.26 ± 0.1	2.54 ± 1.5	32.18 ± 5.5	114.32 ± 77.3	53.77 ± 11.6
120	0.36 ± 0.2	3.27 ± 1.6	32.10 ± 5.2	124.85 ± 75.0	58.54 ± 12.5
130	0.41 ± 0.2	3.79 ± 2.0	31.46 ± 4.9	131.95 ± 95.7	63.35 ± 13.4
140	0.48 ± 0.3	4.46 ± 2.3	31.28 ± 5.1	125.79 ± 94.7	68.15 ± 14.4

Abbreviations: AMT; active motor threshold, MSO; maximum stimulator output, CSP; cortical silent period. All values are mean ± SD. Note that these values represent the grand average of the average of six trials for each stimulus intensity for each participant and the resulting standard deviations.
